# Understanding the Basis of METH Mouth Using a Rodent Model of Methamphetamine Injection, Sugar Consumption, and Streptococcus mutans Infection

**DOI:** 10.1128/mBio.03534-20

**Published:** 2021-03-09

**Authors:** Hiu Ham Lee, Preethi Sudhakara, Shreena Desai, Kildare Miranda, Luis R. Martinez

**Affiliations:** a Department of Biomedical Sciences, NYIT College of Osteopathic Medicine, New York Institute of Technology, Old Westbury, New York, USA; b Department of Oral Biology, University of Florida College of Dentistry, Gainesville, Florida, USA; c University of Miami-Jackson Memorial Hospital Internal Medicine Residency, Miami, Florida, USA; d Instituto de Biofísica Carlos Chagas Filho, Universidade Federal do Rio de Janeiro, Rio de Janeiro, Brazil; e Department of Biological Sciences, The Border Biomedical Research Center, The University of Texas at El Paso, El Paso, Texas, USA; New York University School of Medicine

**Keywords:** biofilms, chlorhexidine, methamphetamine, METH mouth, *Streptococcus mutans*, sucrose

## Abstract

“METH mouth” is a common consequence of chronic methamphetamine (METH) use, resulting in tooth decay and painful oral tissue inflammation that can progress to complete tooth loss. METH reduces the amount of saliva in the mouth, promoting bacterial growth, tooth decay, and oral tissue damage. This oral condition is worsened by METH users’ compulsive behavior, including high rates of consumption of sugary drinks, recurrent tooth grinding, and a lack of frequent oral hygiene. Streptococcus mutans is a Gram-positive bacterium found in the oral cavity and associated with caries in humans. Hence, we developed a murine model of METH administration, sugar intake, and S. mutans infection to mimic METH mouth in humans and to investigate the impact of this drug on tooth colonization. We demonstrated that the combination of METH and sucrose stimulates S. mutans tooth adhesion, growth, and biofilm formation *in vivo*. METH and sucrose increased the expression of S. mutans glycosyltransferases and lactic acid production. Moreover, METH contributes to the low environmental pH and S. mutans sucrose metabolism, providing a plausible mechanism for bacterium-mediated tooth decay. Daily oral rinse treatment with chlorhexidine significantly reduces tooth colonization in METH- and sucrose-treated mice. Furthermore, human saliva inhibits S. mutans colonization and biofilm formation after exposure to either sucrose or the combination of METH and sucrose. These findings suggest that METH might increase the risk of microbial dental disease in users, information that may help in the development of effective public health strategies to deal with this scourge in our society.

## Abstract (precis)

“METH mouth” is characterized by severe tooth decay and gum disease, which often causes teeth to break or fall out. METH users are also prone to colonization by cariogenic bacteria such as Streptococcus mutans.

## INTRODUCTION

Methamphetamine (METH) is an extremely addictive psychostimulant and a major public health problem worldwide (1). The cost of METH abuse in the United States exceeds $30 billion yearly, and its recreational consumption has negative psychological, medical, and social consequences in users (2). It is estimated that 1.6 million Americans use METH each year, and its consumption popularity trend has significantly increased seven or eight times in the last decade (3). METH stimulates the secretion of dopamine in regions of reward in the brain, supporting the user’s compulsive consumption, which results in addiction (4–6). METH is associated with 15% of the all-drug overdose-related deaths in the United States, with half of those deceased involving an opioid (7). METH abuse causes aggression and psychotic behavior, leading users to commit violent crimes (7). Additionally, the intoxicating effects of METH alter judgment and reduce inhibitions, leading people to engage in unsafe activities that are related to risky sexual behavior, resulting in high rates of acquisition of HIV (8) and other transmissible infectious diseases (9–11). These communicable diseases can spread via contaminated needles, syringes, and other equipment shared by multiple METH injection users (8).

A common sign of METH abuse is extreme tooth decay, a condition known as “METH mouth” (12), highly prevalent in prisoners and impacting the U.S. prisons’ budgets. Users with METH mouth have blackened, stained, or rotting teeth, even among young or short-term users (13). The exact causes of METH mouth are not fully understood. The leading hypothesis is that METH constricts blood vessels, thereby limiting the blood supply, resulting in “dry mouth” (xerostomia) (14, 15). A reduction in saliva impairs the mouth’s capacity to neutralize harsh acids produced by oral bacteria after metabolizing carbohydrates, resulting in erosion of the teeth and gums and increasing the susceptibility of teeth to damage (16). This process is exacerbated by behaviors common in users on a METH high: a strong desire for sugary foods and drinks (12), compulsive tooth grinding (bruxism) (17), and neglect of oral care such as brushing and flossing (13). For example, chronic METH users drink on average 35.3 sodas per month (18), brush their teeth less frequently (19), suffer from bruxism (20), and present more dental problems (e.g., tooth decay and periodontal disease, etc.) than nonusers.

We investigated the relationship between METH use, microbial surface colonization, and oral disease using Streptococcus mutans as a model organism. S. mutans is a Gram-positive coccus-shaped bacterium commonly found in the oral cavity and a significant contributor to tooth decay. A well‐characterized, clinically relevant factor in caries development is the ability of S. mutans to metabolize sucrose, the most cariogenic carbohydrate because it can function as a fermentable disaccharide and serve as a substrate for intracellular polysaccharide synthesis (21). Therefore, we hypothesized that the combination of METH and sucrose, due to the consumption of sugary drinks by users, facilitates S. mutans colonization and biofilm formation *in vivo*. Despite substantial clinical evidence associating high rates of sugar consumption, enhanced oral bacterial colonization, and increased tooth decay with oral disease in METH users, there are limited studies investigating the biology of METH mouth.

To our knowledge, this is the first basic science study focused on elucidating the fundamentals of METH mouth using a rodent model of prolonged drug injection and S. mutans oral infection. We aimed to demonstrate that METH administration stimulates sucrose consumption, increases the risk of microbial tooth colonization, and results in oral disease in users. Our findings may have important translational implications for the development of treatments for the management of METH mouth and more effective preventive public health strategies that can be applied to provide effective dental care for METH users in prisons, drug treatment centers, and health clinics. Future implementation of these preventive dental care policies may result in significant economic savings for health care and correctional systems in U.S. regions heavily affected by METH abuse.

## RESULTS

### METH and sucrose stimulate S. mutans replication.

**FIG 1:**
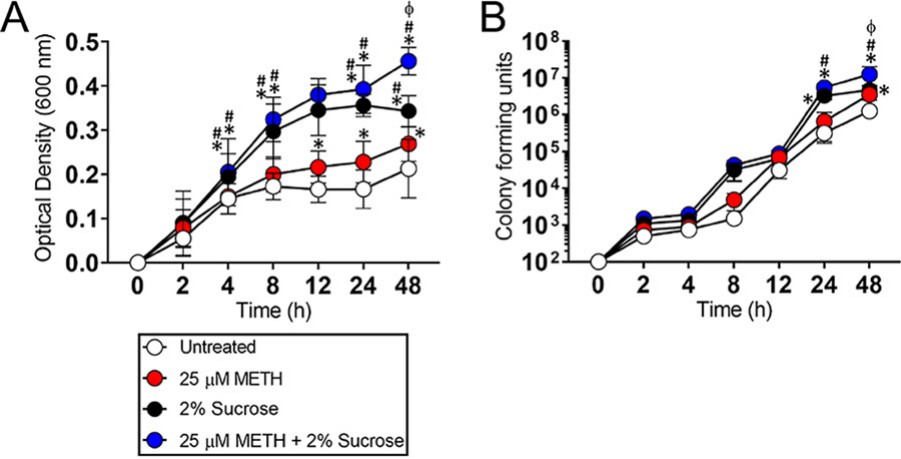
Methamphetamine (METH) and sucrose stimulate S. mutans replication *in vitro*. The effect of METH, sucrose, or their combination on S. mutans growth kinetics was determined via Bioscreen C (A) and CFU (B) analyses. S. mutans was grown in the absence (untreated) or presence of 25 μM METH, 2% sucrose, or 25 μM METH plus 2% sucrose. For real-time spectrophotometry and cell viability assays, each time point represents the average from 16 and 5 individual measurements, respectively. Symbols (*, #, and ϕ) indicate higher proliferation rates than in the untreated, 25 μM METH, or 2% sucrose group, respectively. Each symbol denotes *P* value significance (*P < *0.05) calculated by ANOVA and adjusted by the use of Tukey’s multiple-comparison test. These experiments were performed twice, with similar results obtained each time.

We examined the impact of METH, sucrose, or METH plus sucrose on S. mutans growth in real time for 48 h (Fig. 1A). S. mutans cultured in sucrose or METH plus sucrose demonstrated faster replication than untreated or METH-treated bacteria after 4 h (*P < *0.05). Similarly, METH-treated bacteria showed significantly higher rates of proliferation after 12 h than untreated microbial cells (*P < *0.05). Notably, S. mutans exposed to the combination of METH and sucrose evinced the highest rate of cellular division after 48 h (*P < *0.05). Additionally, we used CFU analysis to validate the results obtained in real time (Fig. 1B). We did not observe any difference in bacterial viability between the groups after 12 h. In contrast, bacteria exposed to METH plus sucrose demonstrated higher viability than microbes in the untreated and METH groups after 24 h (*P < *0.05). S. mutans grown with sucrose also had higher proliferation rates than untreated bacteria after 24 h (*P < *0.05). Finally, bacteria cultured in the presence of METH and sucrose exhibited the highest viability after 48 h (*P < *0.05). Bacteria grown with either METH (*P < *0.05) or sucrose (*P < *0.05) displayed higher proliferation rates than untreated cells after 48 h. Our data reveal that the combination of METH and sucrose promotes S. mutans proliferation and suggest an advantage during oral cavity colonization in the setting of METH use and sugary drink consumption.

### METH and sucrose promote viable S. mutans adhesion to a plastic surface.

**FIG 2:**
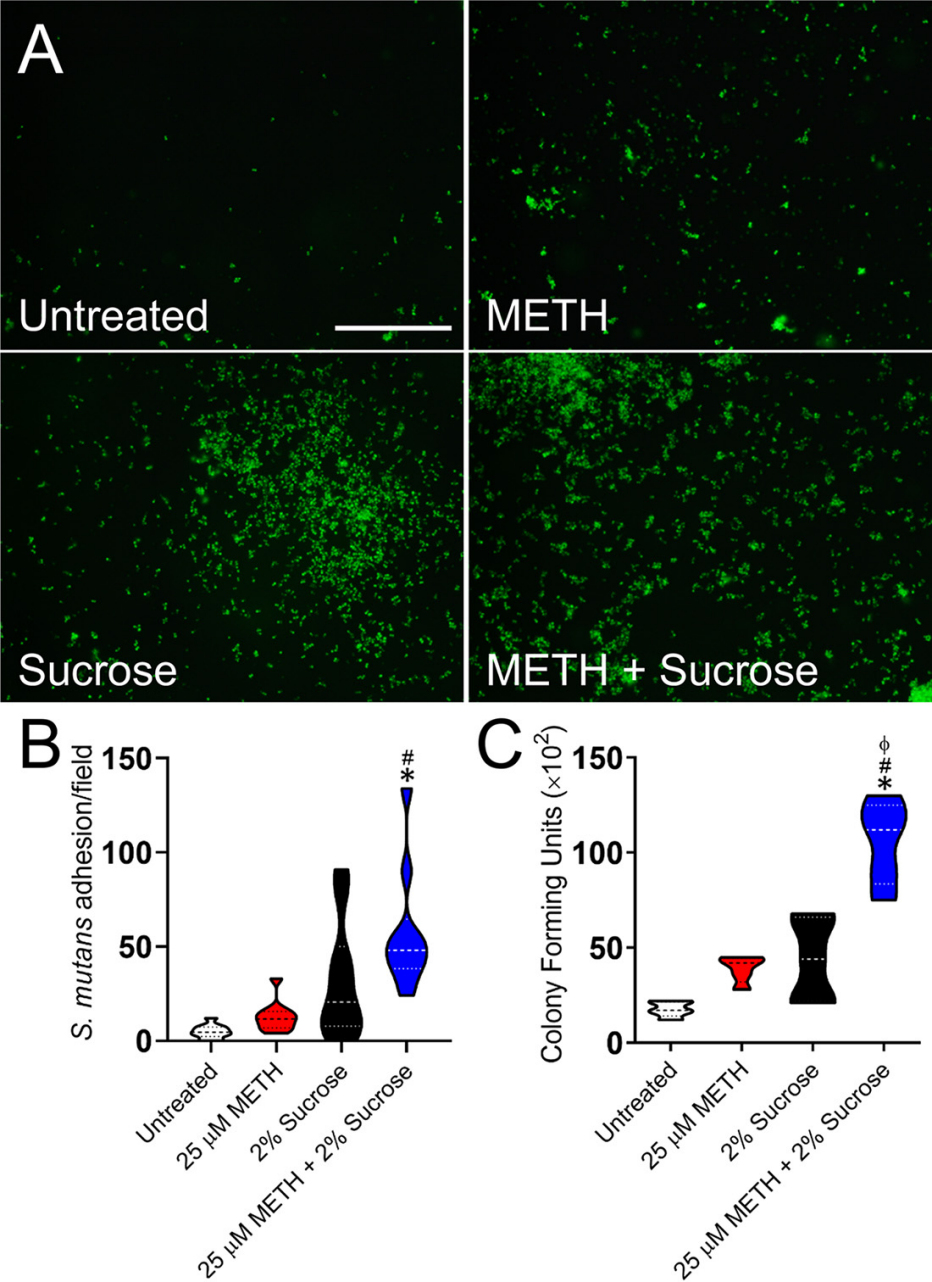
The combination of METH and sucrose promotes adherence of viable Streptococcus mutans to a plastic surface after 4 h of incubation. Bacteria were grown in the absence (untreated) or presence of 25 μM METH, 2% sucrose, and 25 μM METH plus 2% sucrose for 4 h at 37°C in a 5% CO_2_ aerobic atmosphere. Next, S. mutans adhesion to the polystyrene substrate was determined by fluorescence microscopy (A), measurements of bacterial adhesion per field (*n *= 10 replicates under each condition) (B), and CFU counts (*n *= 6 replicates under each condition) (C). For panels B and C, violin plots denote the averages (dashed lines) and replicate distributions. Each assay was performed twice independently, and all the replicates were included in the graphs. Symbols (*, #, and ϕ) indicate high statistical significance compared to the untreated, 25 μM METH, or 2% sucrose group, respectively. Each symbol denotes *P* value significance (*P < *0.05) calculated by ANOVA and adjusted by the use of the Bonferroni correction. These assays were carried out in triplicate and performed twice, with similar results obtained each time.

One of the most important steps in bacterial colonization of the teeth is initial microbial attachment (22). Hence, we evaluated the impact of 25 μM METH, 2% sucrose, and the combination of 25 μM METH plus 2% sucrose on S. mutans adhesion to the wells of polystyrene microtiter plates after incubation at 37°C for 4 h. Using fluorescence microscopy, we observed high-density bacterial adhesion in S. mutans grown in the presence of sucrose (Fig. 2A, bottom left) or METH plus sucrose (bottom right) compared to untreated cells (top left) or cells in the presence of METH alone (top right). We counted the number of bacteria per field that bound to the plastic surface and showed that S. mutans cells incubated with METH and sucrose had significantly higher rates of attachment to polystyrene than untreated and METH-treated bacteria (*P < *0.05) (Fig. 2B). Although S. mutans exposed to METH plus sucrose also showed a trend of increasing adhesion to plastic compared to bacteria cultured with sucrose alone, this tendency was not statistically significant (Fig. 2B). Nevertheless, we recovered significantly high CFU only in S. mutans cultures treated with the combination of METH plus sucrose relative to the other experimental groups (*P < *0.05) (Fig. 2C). Our results suggest that the combination of METH and sucrose promotes initial S. mutans adhesion to a polystyrene surface.

### METH and sucrose facilitate S. mutans biofilm formation *in vitro*.

**FIG 3:**
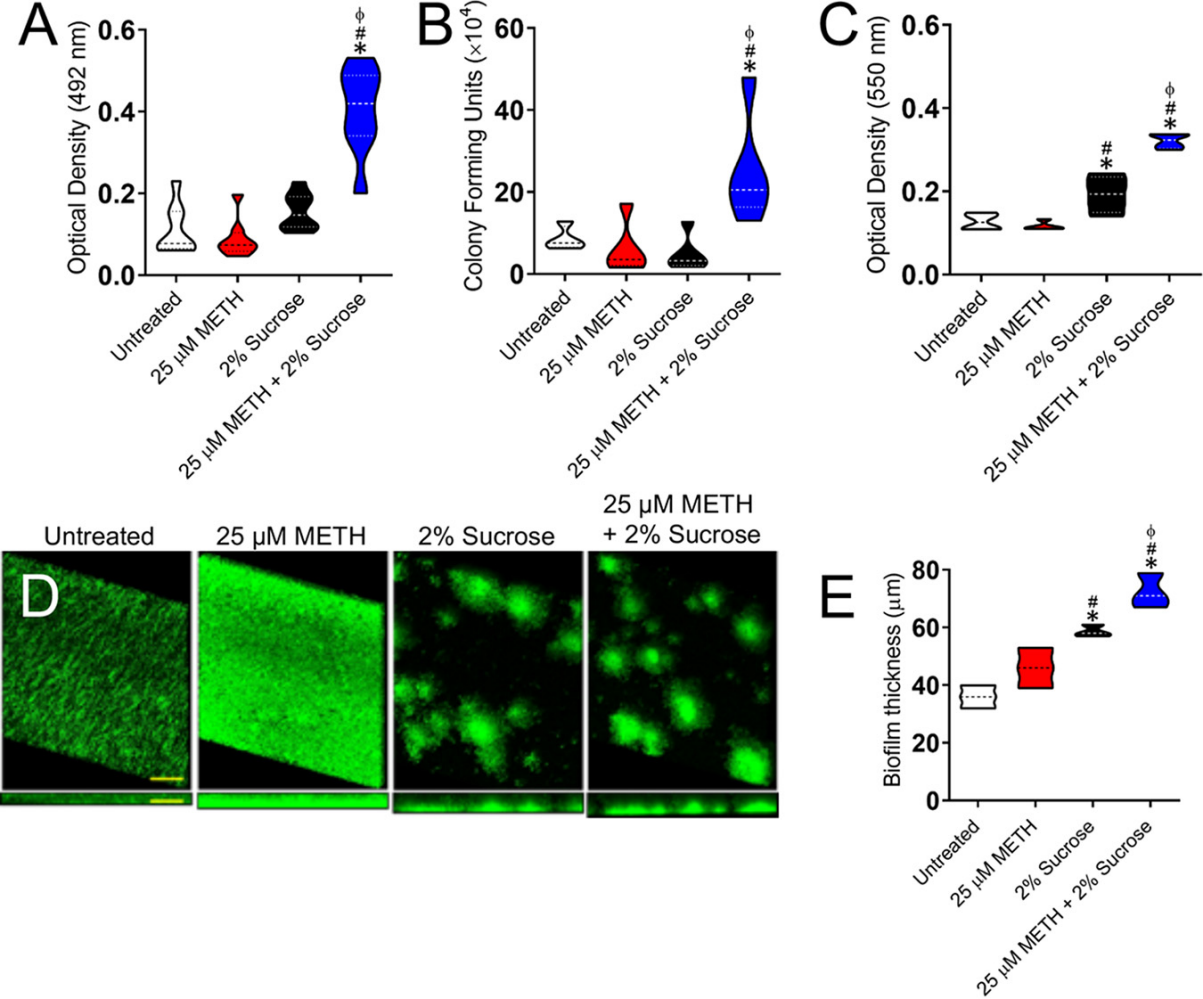
The combination of METH and sucrose enhances S. mutans biofilm formation *in vitro*. (A to C) S. mutans biofilm formation on polystyrene microtiter plates was evaluated by an XTT reduction assay (A), CFU determinations (B), and crystal violet staining (C) after incubation of the bacteria with phosphate-buffered saline (PBS) (untreated), 25 μM METH, 2% sucrose, and 25 μM METH plus 2% sucrose for 48 h at 37°C in a 5% CO_2_ aerobic atmosphere. Violin plots denote the averages (dashed lines) and replicate distributions (*n *= 8 under each condition). All these assays were carried out in quadruplicates and performed twice independently, and all the replicates were included in the graphs. (D) Confocal microscopy of mature S. mutans biofilms formed on glass-bottom plates after incubation of the bacteria (green [SYTO 9]) alone (PBS) (untreated) or with 25 μM METH, 2% sucrose, and 25 μM METH plus 2% sucrose for 48 h at 37°C. The pictures were taken at a magnification of ×63. Bars, 100 μm. (E) The thickness of the bacterial biofilms grown under these conditions was measured by *z*-stack reconstruction. Violin plots show the averages (dashed lines) and distributions from three independent thickness measurements. For panels A to C and E, symbols (*, #, and ϕ) indicate higher statistical significance than in the untreated, 25 μM METH, or 2% sucrose group, respectively. Each symbol denotes *P* value significance (*P < *0.05) calculated by ANOVA and adjusted by the use of Tukey’s multiple-comparison test.

S. mutans belongs to a group of colonizers of human teeth and can metabolize various carbohydrates into organic acids, which may lead to the cariogenic destruction of tooth surfaces (23). Therefore, we investigated the effect of METH, sucrose, or the combination of both on S. mutans biofilm formation *in vitro* (Fig. 3). Using the colorimetric XTT {2,3-bis-(2-methoxy-4-nitro-5-sulfophenyl)-5-[(phenylamino)carbonyl]-2H-tetrazolium hydroxide} reduction assay (Fig. 3A) and CFU counts (Fig. 3B), we found that the combination of 25 μM METH and 2% sucrose significantly increased the metabolic activity (*P < *0.05) and number (*P < *0.05) of biofilm-derived bacteria, respectively, on the plastic surface compared to untreated cells and cells treated with METH or sucrose alone (Fig. 3A and B). Crystal violet staining, which measures both the cellular and extrapolymeric matrix (EPM) components of a biofilm, confirmed that the combination of 25 μM METH and 2% sucrose substantially increased biofilm formation (*P < *0.05) relative to the other conditions (Fig. 3C). However, bacteria grown in the presence of 2% sucrose also exhibited higher rates of biofilm formation than untreated and METH-treated bacteria (*P < *0.05). Confocal microscopy images of mature streptococcal biofilms on glass-bottom plates were analyzed to visualize their architecture and determine their thickness (Fig. 3D). There were variations in the biofilm morphologies showcased by bacteria grown under the different conditions. Streptococci incubated in the absence (untreated) or presence of METH displayed uniform biofilms across the field (Fig. 3D). Although METH-treated biofilms appeared to show higher densities of bacteria likely surrounded by massive amounts of EPM, the average sizes of both untreated (36-μm) and METH-treated (46-μm) biofilms were not statistically significant (Fig. 3E). In contrast, bacteria incubated with sucrose or METH plus sucrose exhibited scattered clumps of cells likely surrounded by vast amounts of EPM throughout the field in a dome-shaped biofilm arrangement (Fig. 3D). S. mutans cells grown with METH plus sucrose developed the thickest (72-μm) biofilms compared to those grown under the other conditions (*P < *0.05) (Fig. 3E). Moreover, microbial cells incubated with sucrose alone formed thicker (59-μm) biofilms than untreated (*P < *0.05) or METH-treated (*P < *0.05) bacteria. These findings indicate that METH and sucrose enhance cell adhesion, metabolic activity, and biofilm formation. In addition, the sucrose and METH plus sucrose conditions promote scattered S. mutans biofilm formation on polystyrene plates.

### METH-injected mice drink significant amounts of water supplemented with sucrose.

**FIG 4:**
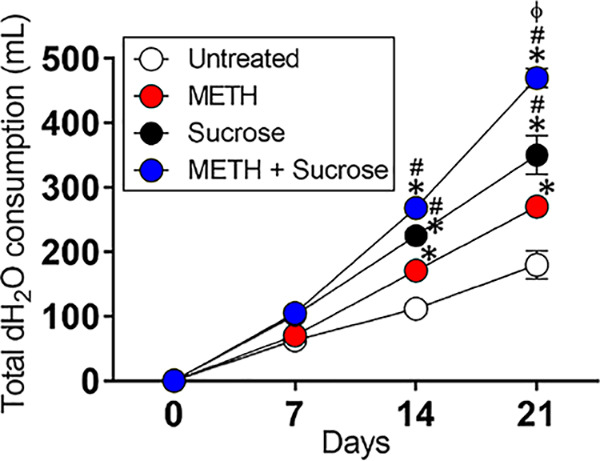
The combination of METH and sucrose increases water (H_2_O) consumption by C57BL/6 mice. The animals’ total H_2_O consumption after 21 days of METH injection and sucrose administration is shown. Mice were daily injected intraperitoneally with PBS (untreated) or METH (2.5, 5, and 10 mg/kg/day on weeks 1, 2, and 3, respectively). In addition, two groups of mice, PBS and METH treated, were supplemented with 2% sucrose in the drinking H_2_O. H_2_O consumption by rodents (*n *= 5 per cage per group) was monitored and recorded every 7 days during 21 days of treatments. Each time point represents three independent measurements. Symbols (*, #, and ϕ) indicate higher H_2_O consumption rates than in the untreated, 25 μM METH, or 2% sucrose group, respectively. Each symbol denotes *P* value significance (*P < *0.05) calculated by ANOVA and adjusted by the use of Tukey’s multiple-comparison test. These experiments were performed twice, with similar results obtained each time.

Since METH may induce cravings for sugary carbonated beverages by users and this behavior has been associated with METH mouth (18), we investigated whether or not the drug stimulates the consumption of water alone or water supplemented with 2% sucrose by injected C57BL/6 mice for 21 days (Fig. 4). There were no differences in water consumption among the tested groups of mice after 7 days. METH-treated mice (170 ml) showed higher water consumption rates than untreated animals (112.5 ml) after 14 days (*P < *0.05). Rodents injected with phosphate-buffered saline (PBS) (untreated) (225 ml) or METH (268 ml) and supplemented with 2% sucrose demonstrated similarly increased water consumption and much higher consumption rates than the other groups after 14 days (*P < *0.05). However, animals treated with METH and supplemented with 2% sucrose (468 ml) in their water displayed significantly higher water intake than the other groups (untreated, 182.5 ml; METH, 270 ml; sucrose, 350 ml) after 21 days (*P < *0.05). These observations indicate that METH-treated C57BL/6 mice take in considerable amounts of water supplemented with sucrose after 21 days. These results also suggest that our murine model of prolonged METH administration is acceptable to investigate the basis of METH mouth.

### METH and sucrose increase S. mutans biofilms on the teeth of C57BL/6 mice.

**FIG 5:**
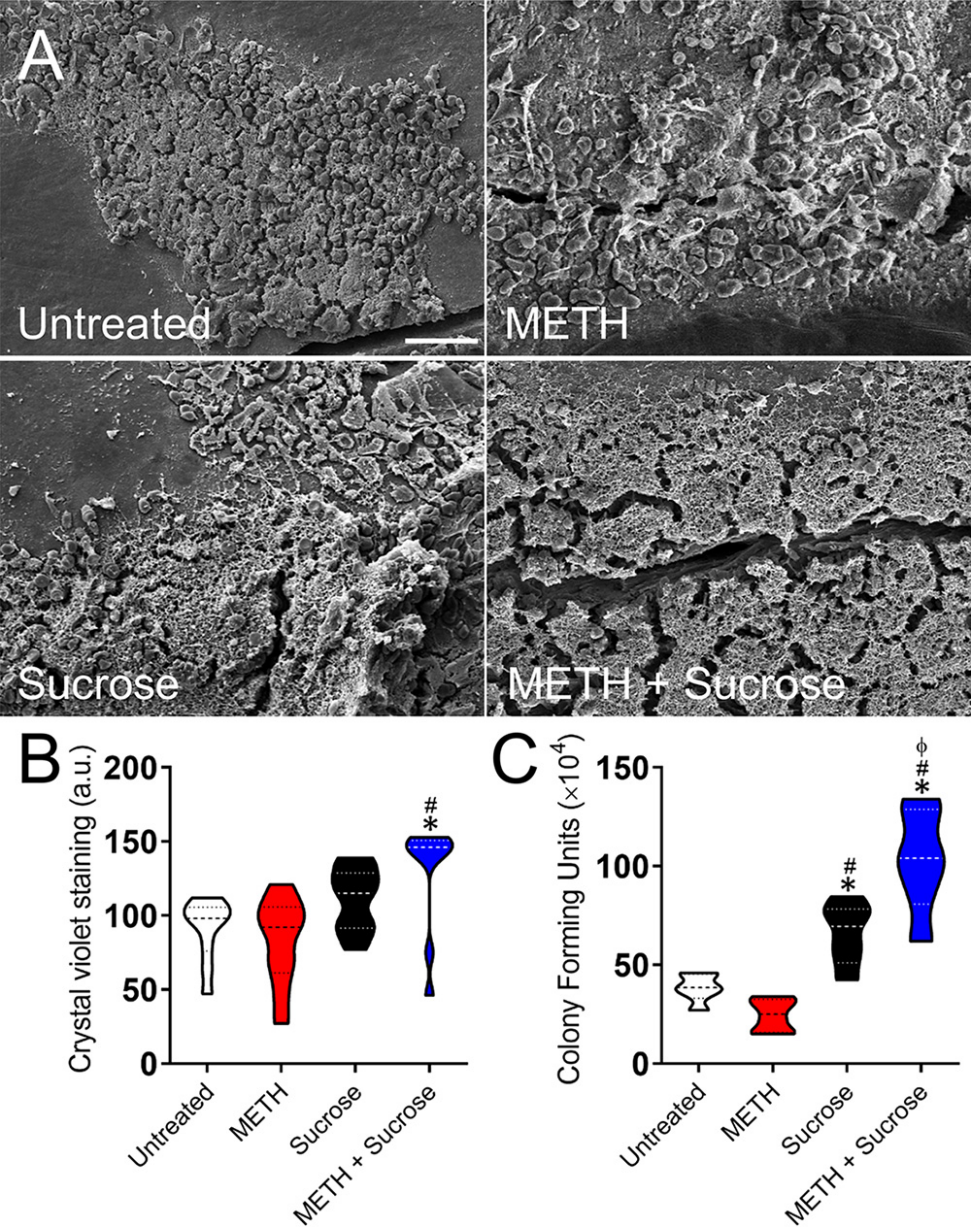
The combination of METH and sucrose increases S. mutans biofilm formation on the teeth of C57BL/6 mice. Shown are scanning electron microscopy (SEM) images of mature S. mutans biofilms formed on the teeth of C57BL/6 mice for 24 h. After METH and sucrose treatments, mice were infected orally with 10^7^ S. mutans bacteria and sacrificed after 24 h, and their frontal teeth were carefully extracted for imaging (bar, 20 μm) (A), crystal violet staining (B), and CFU determinations (C). For panels B and C, violin plots signify the averages (dashed lines) and subject distributions (*n *= 10 for crystal violet staining and *n *= 6 for CFU determinations per group) under each experimental condition. Each assay was performed twice independently, and all the animals for each experiment were included in the graphs. Symbols (*, #, and ϕ) indicate higher statistical significance than in the untreated, 25 μM METH, or 2% sucrose group, respectively. Each symbol denotes *P* value significance (*P < *0.05) calculated by ANOVA and adjusted by the use of Tukey’s multiple-comparison test. a.u., arbitrary units.

Using a prolonged METH administration (24) and S. mutans oral infection mouse model, we investigated the effect of METH, sucrose, or the combination of both on streptococcal biofilm formation *in vivo* (Fig. 5). Scanning electron microscopy (SEM) was used to visualize architectural differences of streptococcal biofilms formed on the teeth of untreated or METH-, sucrose-, or METH- and sucrose-treated mice (Fig. 5A to D). Untreated animals showed biofilms that covered a small area of the tooth, which consisted of localized bacteria surrounded by moderate amounts of EPM (Fig. 5A, top left). The teeth of METH-treated mice displayed scattered streptococci with minimal EPM surrounding the bacteria (Fig. 5A, top right). In contrast, the teeth of sucrose-treated mice exhibited considerable numbers of localized bacteria surrounded by vast amounts of EPM (Fig. 5A, bottom left). Notably, rodents treated with the combination of METH and sucrose demonstrated a dense network of streptococci enclosed in abundant amounts of EPM uniformly distributed throughout the field (Fig. 5A, bottom right). To confirm the SEM findings, we performed crystal violet staining directly on the teeth of each group of mice 24 h after S. mutans infection (Fig. 5B). We did not observe differences in streptococcal biofilm formation between the untreated and METH- or sucrose-treated mice. However, there was a trend toward an increase in the staining of bacterial biofilms on the teeth of sucrose-treated rodents. Animals treated with the combination of METH and sucrose evinced a significantly higher biofilm biomass than untreated (*P < *0.05) and METH-treated (*P < *0.05) mice. Both the sucrose and METH plus sucrose groups showed no difference in biofilm formation. Furthermore, we quantified the number of bacteria on dental biofilms and found that METH- and sucrose-treated mice showed the highest bacterial burden relative to the other conditions (*P < *0.05) (Fig. 5C). Mice treated with sucrose alone also exhibited higher CFU than did the untreated and METH-treated groups (*P < *0.05). Our data demonstrate that sucrose and METH plus sucrose treatments stimulate bacterial colonization and biofilm formation on murine teeth.

### METH and sucrose mediate S. mutans adhesion *in vivo*.

**FIG 6:**
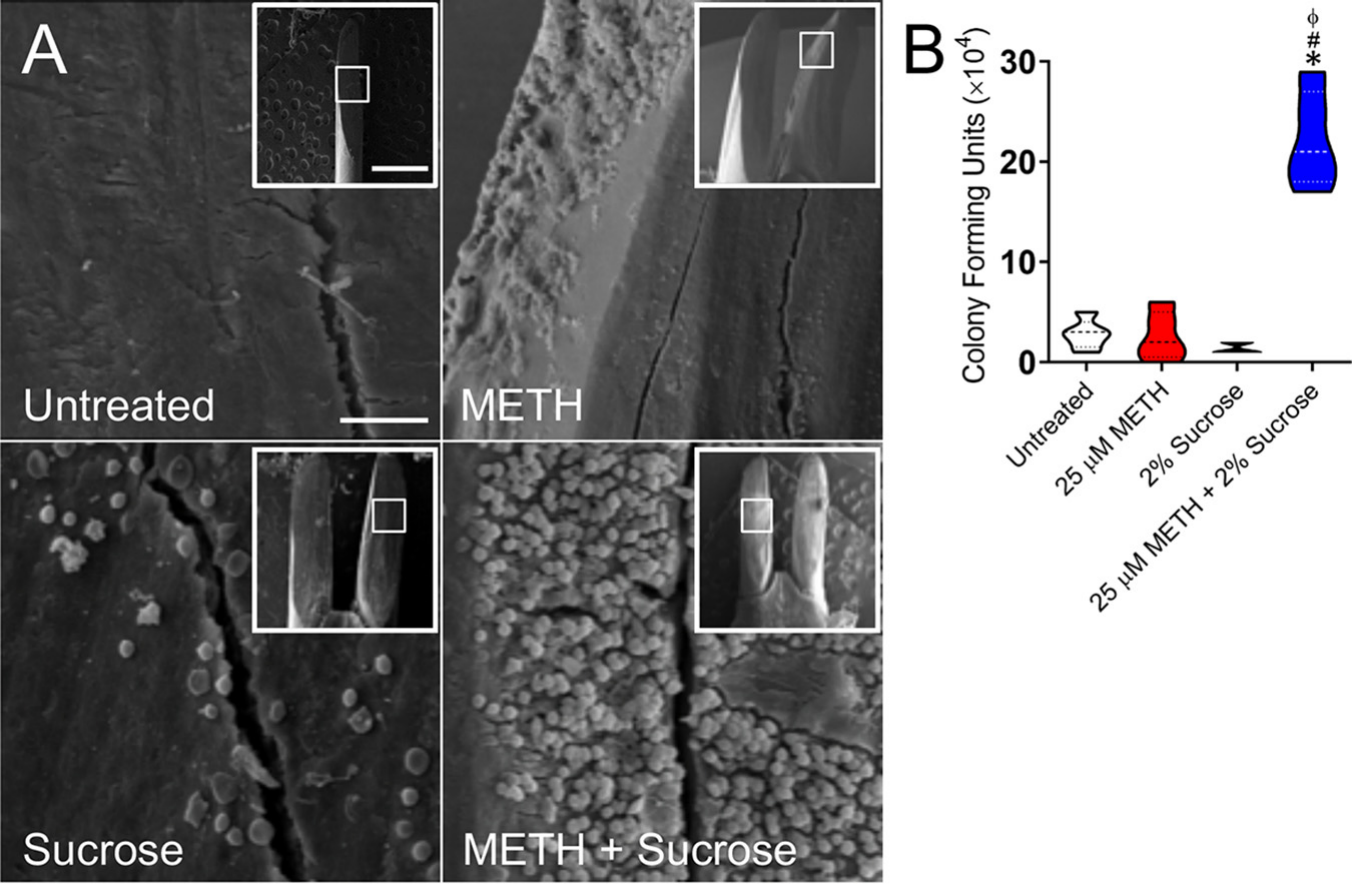
The combination of METH and sucrose promotes S. mutans tooth adhesion in C57BL/6 mice. (A) SEM images of streptococcal cells adhered on the teeth of C57BL/6 mice after 4 h of oral infection with 10^7^ bacteria. Bars, 10 μm and 1 mm (inset). (B) Attachment of bacteria to the teeth of untreated animals or animals treated with METH, 2% sucrose, and METH plus 2% sucrose after 4 h of oral infection was evaluated by counting CFU. Violin plots indicate the averages (dashed lines) and distributions of the results for five animals per group. Symbols (*, #, and ϕ) indicate higher adhesion than in the untreated, 25 μM METH, or 2% sucrose group, respectively. Each symbol denotes *P* value significance (*P < *0.05) calculated by ANOVA and adjusted by the use of Tukey’s multiple-comparison test.

The initial physical attraction of bacteria and adhesion to abiotic or biotic surfaces are critical for biofilm formation and maturation (25, 26). Since this process has important implications for microbial pathogenesis, we investigated the effect of METH, sucrose, or the combination of both on the attachment of streptococci to the dental surface of mice after a 4-h oral infection using SEM and CFU counts (Fig. 6). SEM images demonstrated minimal bacterial attachment to the dental surface of untreated mice (Fig. 6A, top left) and their METH (top right)- or sucrose (bottom left)-treated counterparts. The teeth of animals treated with METH plus sucrose exhibited a substantial number of streptococci adhered and uniformly distributed throughout the field without EPM (Fig. 6A, bottom right). These results were confirmed by CFU determinations showing a highly significant bacterial burden in mice treated with METH and 2% sucrose compared to the other groups (*P < *0.05) (Fig. 6B). We observed that the combination of METH and sucrose supports S. mutans colonization of C57BL/6 mouse teeth.

### METH and sucrose augment S. mutans glucosyltransferase expression.

**FIG 7:**
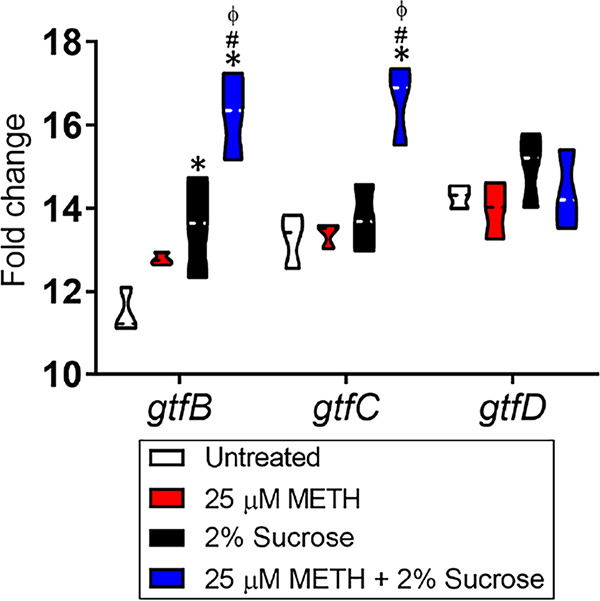
The combination of METH and sucrose induces the expression of the S. mutans glucosyltransferase genes *gtfB* and *gtfC*. The differential expression of S. mutans glucosyltransferase-encoding genes (*gtfB*, *gtfC*, and *gtfD*) was measured using reverse transcriptase PCR. Bacteria were cultured in the absence or presence of 25 μM METH, 2% sucrose, or the combination for 24 h. 16S rRNA was used as the housekeeping gene control. Violin plots represent the averages and distributions from three independent measurements in triplicates. Symbols (*, #, and ϕ) indicate significantly higher expression levels than in the untreated, 25 μM METH, or 2% sucrose group, respectively. Each symbol denotes *P* value significance (*P < *0.05) calculated by ANOVA and adjusted by the use of Tukey’s multiple-comparison test.

Biofilm formation in S. mutans is promoted by major virulence factors known as glucosyltransferases, which synthesize adhesive extracellular polysaccharides utilizing dietary sucrose (27). Hence, we examined the impact of METH, sucrose, or the combination of both on the expression of three S. mutans glucosyltransferases (*gtfB*, *gtfC*, and *gtfD*) using real-time PCR (RT-PCR) analysis after 24 h of incubation (Fig. 7). *gtfB* binds to other oral bacteria, promoting the formation of polymicrobial biofilms, whereas *gtfC* enables S. mutans to stick to the pellicle on the tooth enamel (23). *gtfB* and *gtfC* were significantly increased in bacteria incubated in the presence of 25 μM METH plus 2% sucrose compared with the other conditions (*P < *0.05). Likewise, streptococci grown with sucrose showed higher expression levels of *gtfB* than did untreated bacteria (*P < *0.05). Although *gtfD* produces soluble glucans that serve as primers for *gtfB* to synthesize more extracellular polysaccharides, we did not find any difference between the tested groups. In summary, we showed that METH plus sucrose treatment increases the expression of S. mutans *gtfB* and *gtfC*.

### Environmental pH reduction results from S. mutans sucrose metabolism and METH acidity.

**FIG 8:**
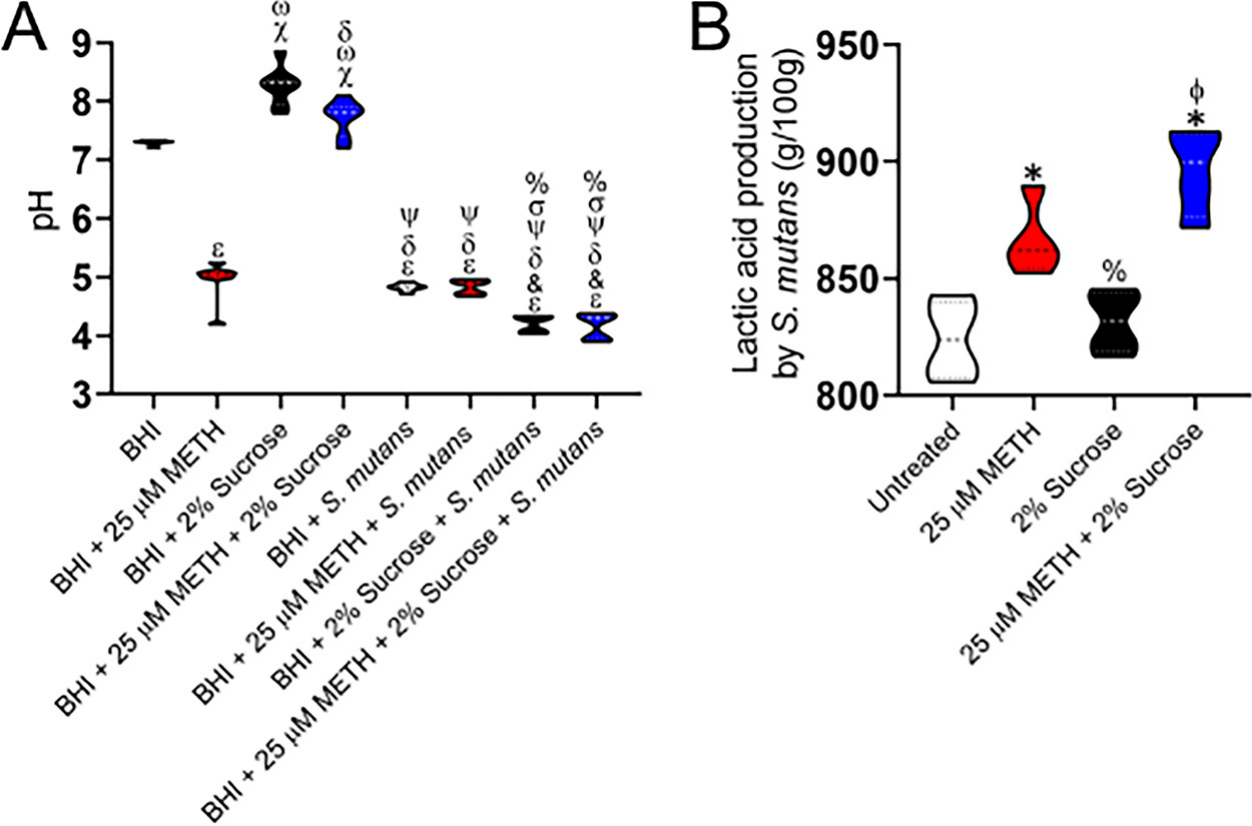
Sucrose promotes S. mutans environmental pH reduction. (A) Changes in brain heart infusion (BHI) broth pH by S. mutans were measured using a pH meter. The following conditions were tested: BHI broth alone or supplemented with 25 μM METH, 2% sucrose, or METH plus 2% sucrose in the absence or presence of S. mutans. Violin plots indicate the averages (dashed lines) and distributions from eight independent measurements. Each symbol (ε, &, δ, ψ, σ, %, χ, and ω) denotes *P* value significance (*P < *0.05) calculated by ANOVA and adjusted by the use of Tukey’s multiple-comparison test. ε, &, δ, ψ, σ, and % indicate significantly lower pH than in the BHI broth, BHI broth plus 25 μM METH, BHI broth plus 2% sucrose, BHI broth plus 25 μM METH and 2% sucrose, BHI broth plus S. mutans, and BHI broth plus 25 μM METH and S. mutans groups, respectively. χ and ω denote significantly higher pH than in the BHI broth and BHI broth plus 25 μM METH groups, respectively. (B) Lactic acid production by S. mutans was quantified after incubation in the absence (untreated) or presence of 25 μM METH, 2% sucrose, or METH plus 2% sucrose. Violin plots indicate the averages (dashed lines) and distributions from four measurements. Each symbol (*, ϕ, and %) denotes *P* value significance (*P < *0.05) calculated by ANOVA and adjusted by the use of Tukey’s multiple-comparison test. * and ϕ indicate significantly higher lactic acid production than in the untreated and 2% sucrose groups, respectively. % denotes significantly lower lactic acid synthesis than in the 25 μM METH group. Both assays were performed twice independently, and all the replicates are shown in each graph.

S. mutans is an acidogenic microbe that metabolizes and ferments dietary carbohydrates into lactic acid, reducing the overall pH of the oral environment and promoting tooth decay (27). Thus, we investigated the impact of S. mutans, METH, sucrose, or the combination on the pH of the growth medium. In this regard, the pH of brain heart infusion (BHI) broth alone or supplemented with 25 μM METH, 2% sucrose, or 25 μM METH plus 2% sucrose was measured in the absence or presence of S. mutans using a calibrated pH meter after 48 h of incubation at 37°C (Fig. 8A). As expected from the manufacturer’s specifications, the pH of BHI broth was 7.31. In contrast, adding METH to the medium considerably dropped the pH to 4.96, which is indicative that the drug has an important effect on the acidity of the medium. The addition of sucrose to BHI broth resulted in the highest alkaline pH (8.27) among the samples (*P < *0.05). However, BHI broth supplemented with both METH and sucrose evinced a higher pH (7.72) than the medium alone or with the drug (*P < *0.05). S. mutans grown in BHI broth or BHI broth with METH similarly reduced the medium pHs to 4.82 and 4.84, respectively. These two conditions also demonstrated a significantly lower pH than with BHI broth alone or supplemented with sucrose or the combination (*P < *0.05). Likewise, bacteria cultured with BHI broth with either sucrose (pH 4.21) or a combination of the drug and polysaccharide (pH 4.17) resulted in significant acidity of the medium relative to all the other conditions (*P < *0.05). Given these results, we assessed the effect of METH, sucrose, or the combination on lactic acid synthesis by S. mutans to validate the causes of the acidic medium milieu (Fig. 8B). S. mutans grown with METH showed significantly higher lactic acid levels than untreated bacteria (*P < *0.05). Although not statistically significant, METH-treated bacteria had an increasing lactic acid synthesis trend compared to microbial cells grown in the presence of sucrose. S. mutans cultured with METH and sucrose demonstrated the highest level of lactic acid production (*P < *0.05), suggesting the importance of both metabolites for the acidic pH milieu observed in our studies. These results reveal the influence of METH on reducing the environmental pH and promoting S. mutans sucrose metabolism and growth.

### Daily oral rinse treatment prevents S. mutans tooth colonization.

**FIG 9:**
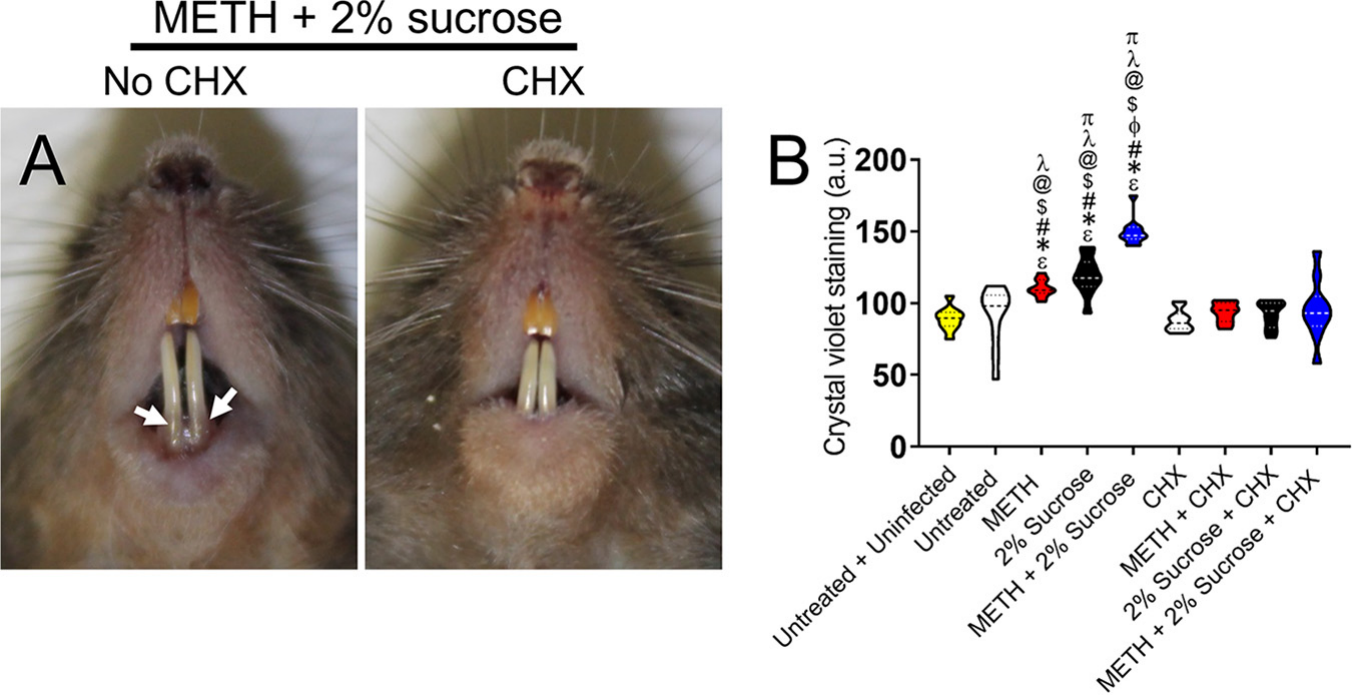
Daily mouth rinse of C57BL/6 mice infected with S. mutans reduces bacterial tooth colonization. (A) Photographs of C57BL/6 mice injected with METH, supplemented with 2% sucrose in the drinking H_2_O, infected with 10^7^ S. mutans, and treated daily with either PBS (no chlorhexidine [CHX]) or CHX for 7 days. White arrows denote crystal violet staining (purple) as indicative of S. mutans tooth colonization and biofilm formation. (B) Crystal violet staining was used to quantify the biofilm biomass on the teeth of mice at 7 days postinfection. The following groups were tested: untreated and uninfected, untreated, METH, 2% sucrose, METH plus 2% sucrose, CHX, METH plus CHX, 2% sucrose plus CHX, and METH plus 2% sucrose and CHX. Violin plots indicate the averages (dashed lines) and distributions from 10 independent measurements (2 teeth per mouse; *n *= 5). Symbols (ε, *, #, ϕ, $, @, λ, and π) indicate significantly higher staining than in the untreated and uninfected, untreated, METH, 2% sucrose, CHX, METH plus CHX, 2% sucrose plus CHX, and METH plus 2% sucrose and CHX groups, respectively. Each symbol denotes *P* value significance (*P < *0.05) calculated by ANOVA and adjusted by the use of Tukey’s multiple-comparison test.

The propensity of S. mutans to form oral biofilms allows it to persist and survive on these abiotic surfaces for long periods, resulting in plaque formation and tooth cavities (28). METH use and consumption of sugary beverages have been associated with METH mouth (12, 18). Chlorhexidine (CHX) is a cationic compound that interacts with the negative charges of bacterial cell walls, resulting in destabilization of the cytoplasmic membranes (29). Hence, we examined the efficacy of daily CHX mouth rinse treatment in reducing or eradicating cariogenic S. mutans biofilms grown on the teeth of C57BL/6 mice using crystal violet staining (Fig. 9). Photographs of rodents injected with METH, supplemented with 2% sucrose in H_2_O, and treated daily with PBS (no CHX) (left) or CHX (right) oral rinse are shown in Fig. 9A. Mice injected with METH that drank water supplemented with 2% sucrose evinced the highest S. mutans biofilm biomass (Fig. 9B). Similarly, animals administered METH or 2% sucrose also showed a more significant S. mutans biofilm biomass than all the other groups (*P < *0.05) (Fig. 9B). Interestingly, mice in the groups administered METH, 2% sucrose, or the combination of METH and 2% sucrose treated daily with CHX mouth rinse demonstrated reduced S. mutans biofilm biomass compared to those of similarly treated groups without CHX (Fig. 9B). These findings demonstrate that daily treatment with CHX oral rinse aids in reducing S. mutans biofilm biomass on the teeth of C57BL/6 mice.

### Human saliva decreases S. mutans biofilm formation *in vitro*.

**FIG 10:**
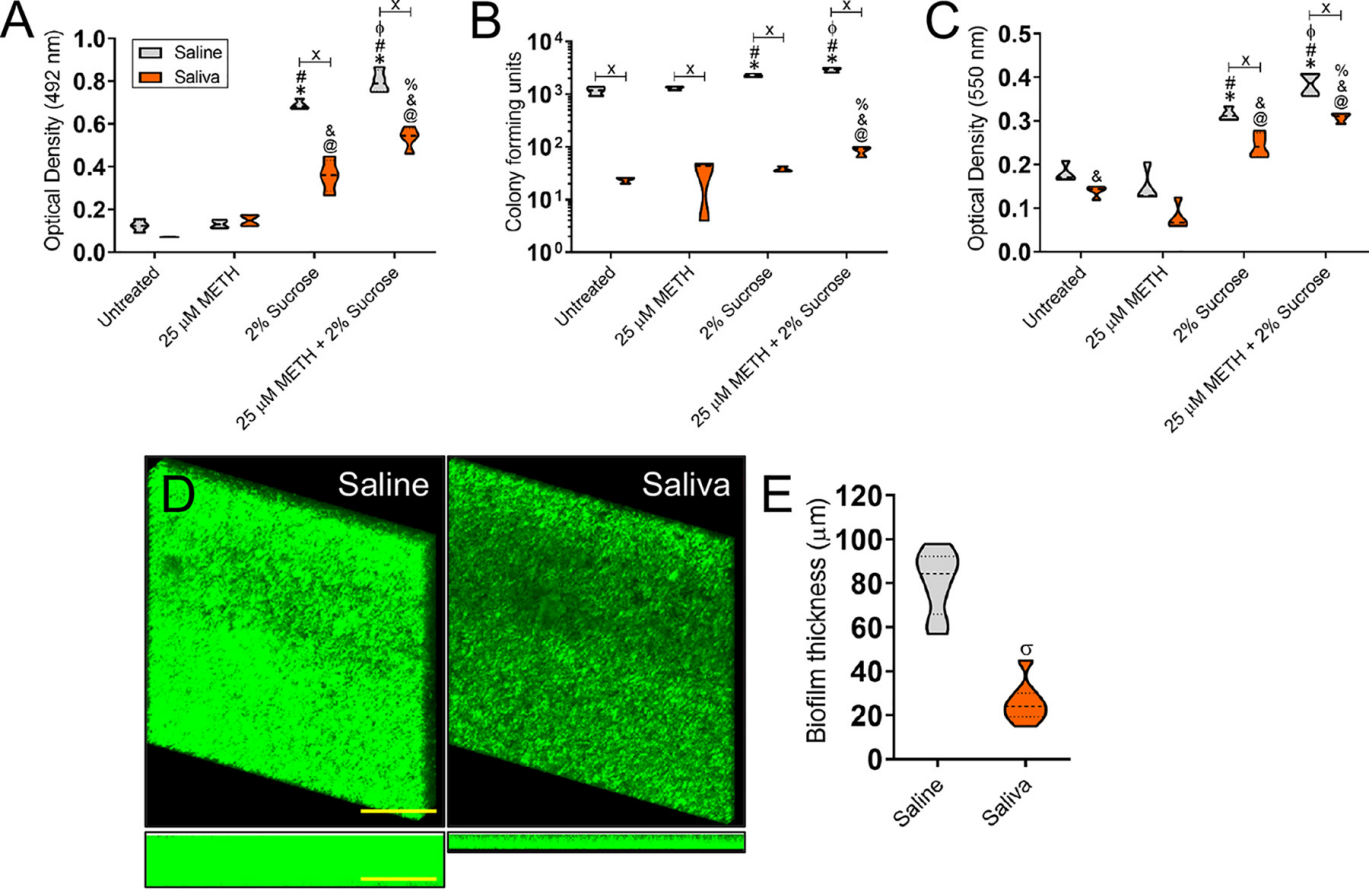
Surface preconditioning with human saliva considerably reduces S. mutans biofilm formation *in vitro*. (A to C) Biofilm formation on 96-well microtiter plates was determined by an XTT reduction assay (A), CFU determination (B), and crystal violet staining (C) after 1 h of preconditioning of the plastic surface with 100 μl of PBS or saliva and incubation of bacteria with PBS (untreated), 25 μM METH, 2% sucrose, and 25 μM METH plus 2% sucrose for 48 h at 37°C in a 5% CO_2_ aerobic atmosphere. Violin plots indicate the averages (dashed lines) and replicate distributions (*n *= 8 under each condition). All these assays were carried out in quadruplicates under each condition and performed twice independently, and all replicates were included in each graph. Symbols for the saline (*, #, and ϕ) and saliva (@, &, and %) conditions indicate significantly higher values than in the untreated, METH, 2% sucrose, and METH plus 2% sucrose groups, respectively. Each symbol denotes *P* value significance (*P < *0.05) calculated by ANOVA and adjusted by the use of Tukey’s multiple-comparison test. Crosses (saline versus saliva) indicate *P* value significance (*P < *0.05) calculated using Student’s *t* test. (D) Confocal microscopy of mature S. mutans biofilms formed on preconditioned glass-bottom plates with saline or saliva after incubation of the bacteria (green [SYTO 9]) with 25 μM METH plus 2% sucrose for 48 h at 37°C. The pictures were taken at a magnification of ×63. Bars, 100 μm. (E) The thickness of the streptococcal biofilms grown under these conditions was measured by *z*-stack reconstruction. Violin plots represent the averages and distributions from three independent measurements. The sigma symbol denotes *P* value significance (*P < *0.05) calculated by Student’s *t* test.

The dental effects of long-term METH use are often attributed to its impact on reducing saliva (16). METH causes dry mouth, which has been associated with cariogenic bacterial proliferation and colonization of the teeth (14, 15). We assessed the effect of human saliva on preventing S. mutans abiotic surface colonization and biofilm formation (Fig. 10). We preconditioned the wells of polystyrene microtiter plates with either PBS (saline) or human saliva for 1 h at room temperature. Next, a suspension of S. mutans on medium alone (untreated) or supplemented with either 25 μM METH, 2% sucrose, or the combination of 25 μM METH and 2% sucrose was added to the solid surface and incubated for 24 h at 37°C. Using the XTT assay (Fig. 10A) and crystal violet staining (Fig. 10C), we observed that saliva preconditioning significantly impairs biofilm formation in bacteria grown in the presence of 2% sucrose or 25 μM METH plus 2% sucrose (*P < *0.05). CFU determinations indicated that saliva pretreatment of the plastic surface significantly reduced bacterial viability under all the tested conditions compared to saline pretreatment (*P < *0.05) (Fig. 10B). Confocal microscopy images demonstrated that saliva preconditioning substantially reduces the thickness of S. mutans biofilms (Fig. 10D, right) grown with METH plus sucrose relative to those of bacterial biofilms grown similarly on saline-pretreated surfaces (Fig. 10D, left). Biofilms grown on solid surfaces pretreated with saliva were ∼25 μm in depth, compared to ∼90 μm in depth in those grown in wells pretreated with saline (*P < *0.05) (Fig. 10E). We demonstrated that human saliva is important in preventing S. mutans colonization and biofilm formation after treatment with sucrose or METH plus sucrose.

## DISCUSSION

We investigated the basis of METH mouth and demonstrated that the combination of METH and sucrose stimulates S. mutans proliferation and colonization of the oral cavity using a novel murine model of drug injection, sugar consumption, and oral infection. We documented that METH enhances the intake of sugary water by C57BL/6 mice, and this behavior can be attributed to their increased physical activity, which is mediated by the drug 5 to 10 min after injection (24). The intense euphoria and hyperactivity exhibited by METH-treated mice are caused by increasing levels of dopamine released in the central nervous system (30), and these effects can last for several hours (24), resulting in the animals’ hyperthermia (31), dehydration (15), and, thus, craving for water consumption. We observed that rodents injected with METH drank substantially more water than untreated controls. A previous study using a similar model of METH administration reported the negative impact of this psychostimulant on mouse physical activity and weight loss (24).

METH users have a predilection for soft drink consumption to relieve the sensation of dry mouth (16), an impulsive desire associated with dental decay (12, 18). Sugary beverages such as soda have low pH, and their underlying acidity is linked to tooth erosion (18). The high sugar content and METH-induced low saliva production provide an ideal environment for adhesion, colonization, and biofilm formation by cariogenic oral bacteria such as S. mutans. Our *in vitro* and *in vivo* studies revealed that the combination of METH and sucrose significantly promotes S. mutans adhesion and biofilm formation. Confocal microscopy images revealed that sucrose induces dispersed S. mutans biofilm formation in dome-shaped microcolonies of bacteria likely embedded within an extracellular glycocalyx, with channels and cavities to allow the exchange of nutrients and waste (32). For some soil bacteria (33) and fungi (34, 35), the scattered distribution and presence of channels are required for cell alignment, advancement on surfaces, and polymicrobial interactions (33). Also, the Vibrio cholerae biofilm structure is determined by demarked zones within the biofilm containing bacteria in different phases of growth (36).

Sucrose is the substrate for glucosyltransferase‐mediated, sucrose‐dependent glucan production, which promotes the adhesion of S. mutans to the tooth surface (37). We evinced that *gtfB* and *gtfC* expression levels were high in bacteria incubated with METH plus sucrose, and this increase was related to considerable bacterial adhesion *in vitro* and *in vivo*. These glucosyltransferases bind to the tooth in the oral cavity and rapidly metabolize sucrose, resulting in the synthesis of insoluble and soluble glucans that serve as anchoring sites for S. mutans and other oral microorganisms to adhere to and cluster on the tooth enamel, resulting in dental plaque or multispecies biofilms (38). Future studies examining the role of S. mutans single and double *gtf* mutants in tooth adhesion and oral biofilm formation using our murine model of METH mouth are warranted and will provide details of the molecular mechanisms associated with METH and sucrose consumption. However, studies testing the adhesion of S. mutans *gtfB*, *gtfC*, or double mutant strains to a plastic surface in medium supplemented with sucrose demonstrated 1- to 2-fold reductions in their attachment relative to the wild-type strain (39), indicating that comparable results might be expected in our model. Similarly, the inactivation of *gtf* genes in Streptococcus downei showed that single or double gene mutations impair bacterium adhesion/biofilm formation on polystyrene microtiter plates (40). Sucrose can also be used by S. mutans to generate organic acids that bring about demineralization leading to dental caries (41). We observed that the combination of METH and sucrose facilitates the production of lactic acid, and this metabolism substantially acidifies BHI broth. Interestingly, the addition of METH to the medium without the presence of the bacterium caused a considerable drop in the pH, providing supporting evidence that the chemical and physical nature of METH may have a significant impact on the tooth erosion, decay, and loss documented in users suffering from METH mouth (42, 43). For example, our findings align with those of a study in South Africa on the pH levels of METH samples sold in the streets of Cape Town (44), which revealed that due to the hydrochloride acid used in its synthesis, the average METH sample pH is 5 (range, 3.02 to 7.03) and should cause extensive damage to the tooth enamel, especially in individuals with hyposalivation. Although the direct impact of METH on the mouth pH of users who inject the drug might be minimal, and the low pH of the oral cavity is likely associated with the consumption of carbonated drinks (18) and drug-induced hyposalivation (45), METH users tend to utilize multiple routes to administer the drug, such as smoking, snorting, or swallowing in a pill form, which can have direct consequences in reducing the mouth pH and directly damage the tooth enamel. Another possible effect of METH administration is the alteration of the oral microbiota facilitating growth and colonization of the tooth surface by S. mutans and other acidogenic bacteria. This premise is supported by recent microbiome evidence indicating that METH causes gut dysbiosis, an important observation in the understanding of drug abuse and the treatment of addiction and its collateral damage such as oral disease (46). METH stimulates S. mutans lactic acid production in the absence or presence of sucrose, suggesting that this substance of abuse may play an important role in altering the bacterium metabolism. It is possible that in the absence of sucrose, METH stimulated the consumption of glucose on BHI broth by S. mutans through the bacterial phosphotransferase systems (PTSs) and/or permeases (23). Upon internalization, glucose is phosphorylated, processed to fructose-6-phosphate, and fermented by glycolysis, resulting in the production of organic acids, mainly lactic acid. This explains why S. mutans treated with METH produces higher levels of lactic acid than microbes grown in the presence of sucrose. Similarly, the lower pH seen in S. mutans cultured in BHI broth with sucrose than in bacteria cultured in BHI broth with METH can also be attributed to the production of other organic acids (e.g., formic and acetic acids) accumulating in the medium (47) that could not be detected by the lactic acid determination kit. Due to this limitation, investigations of S. mutans PTS function and carbohydrate metabolism, particularly the production of other organic acids by fermentation, after exposure to METH are necessary to determine the relationship of this substance of abuse, microbial function, and oral disease.

METH enhances S. mutans growth in BHI broth with and without sucrose, which provides an advantage to the bacterium in tooth colonization. Cigarette smoking is correlated with METH use (13, 48), and nicotine also facilitates microbial growth, biofilm formation, and biofilm metabolic activity (49–51). Nicotine also facilitates the coaggregation of S. mutans and the fungus Candida albicans in the presence of sucrose via the upregulation of *gtfB* (52), a possible consequence that, based on our findings, could be further exacerbated by METH, compromising the users’ oral health status. Nevertheless, we demonstrated that daily mouthwashes with CHX reduce S. mutans biofilm formation on the teeth of C57BL/6 mice orally infected with METH, sucrose, or the combination to baseline levels. CHX not only is effective in eliminating and preventing S. mutans colonization of tooth surfaces (53) but also inhibits the action of glucosyltransferases (54) that are substantially produced by the bacterium after murine ingestion of sucrose. Even though it is difficult to implement habitual and simple oral treatments in METH users, this strategy can be applied to controlled at-risk populations such as prisoners, for whom it has been reported that this drug considerably busts correctional health care budgets due to the high costs of dental care (55). We envisioned that a liquid mouthwash or gum containing CHX can be provided to METH users as a preventive method to combat S. mutans oral colonization and tooth damage. For instance, CHX skin cleaning and clothes washing of detainees considerably reduce cutaneous Staphylococcus aureus colonization (56). Additionally, prolonged and daily chewing of xylitol gum prevents the accumulation of S. mutans in plaque (57). These examples suggest that preventive oral care may result in beneficial outcomes for METH users while reducing the cost of dental care within correctional facilities and saving millions of dollars in taxpayer contributions.

Saliva plays a significant role in the prevention of caries. METH causes hyposalivation (45) and lowers the pH and buffering capacity of saliva (58), which prevents microbial tooth colonization, caries, and loss. Our results demonstrate that human saliva significantly prevents S. mutans biofilm formation on polystyrene in cultures in the absence or presence of METH, sucrose, or the combination. Saliva contains antiglucosyltransferase immunoglobulins (59), which are important in neutralizing METH-induced glucosyltransferases responsible for S. mutans adhesion and polymicrobial biofilm formation (60). It is also rich in antimicrobial peptides such as histatin-5 and lysozyme that prevent S. mutans growth and caries in METH users (61). Chewing sugar-free gum is a validated method that can be easily distributed among METH users to stimulate their salivation after drug utilization, maintain their oral hygiene after sugary beverage consumption, neutralize the acidity of the mouth, and, thus, decrease the incidence of oral disease and dental loss (62).

Our data suggest that it is possible to mitigate oral microbial colonization in the setting of METH use through palliative and preventive care. METH mouth is a very complex public health problem exacerbated by multiple behavioral (e.g., oral hygiene), physiological (e.g., hyposalivation), chemical (e.g., METH-associated low pH), and microbiological (e.g., microbial colonization) factors that contribute to a high tooth decay incidence, especially in chronic users. A major difficulty in treating METH users suffering from METH mouth is their elusive nature of seeking dental care and their preference for self-treatment due to their addiction stigma. Therefore, it is important that public health providers have this in mind and develop simple educational information and medical interventions for METH mouth management in these patients, including the distribution of oral hygiene products such as mouthwash solutions and sugar-free chewing gum supplemented with effective microbicides. Although the mouse model might not necessarily reproduce human oral disease precisely, the model presented in this study is a reasonable animal model of METH administration, sugar consumption, and infection that can be used to dissect the biological details of METH mouth. The findings may translate into new knowledge, studies involving humans or human samples, and the development of therapeutic and public health strategies to deal with the devastating complications of METH mouth.

## MATERIALS AND METHODS

### Streptococcus mutans.

S. mutans strain Clarke 25175 was acquired from the American Type Culture Collection and used in all the experiments. The strain was stored at −80°C in brain heart infusion (BHI) broth (Becton, Dickinson [BD]) with 40% glycerol (Sigma) until use. Streptococci were grown in BHI broth for 24 h at 37°C in a 5% CO_2_ aerobic atmosphere. Growth was monitored by measuring the optical density at 600 nm (OD_600_) using a microtiter plate reader (Bio-Tek).

### Rationale for METH doses used in mice and cell culture.

The concentrations of METH used in the experiments are physiologically relevant. Controlled studies have indicated that a single 260-mg dose peaks at a level of 7.5 μM (63). A single dose of 260 mg would be expected to produce 7.5 to 28.8 μM blood METH levels. Binge doses of 260 to 1,000 mg produce 17 to 80 μM blood METH levels and levels in the micromolar range of hundreds in organs (64). Thus, we selected 2.5 to 10 mg of METH/kg of body weight/day to perform our *in vivo* experiments (24) and 25 μM METH to perform our *in vitro* experiments (65).

### Growth curve.

To determine the impact of METH, sucrose, or their combination on S. mutans growth, BHI broth was inoculated with a fresh colony grown on BHI agar plates and suspended in 1 ml of medium. A suspension of 100 μl of S. mutans was transferred to a 200-well plate with 50 μl of BHI broth per well containing 25 μM METH, 2% sucrose, or 25 μM METH plus 2% sucrose. To limit oxygen exposure, an overlay of 50 μl of sterile mineral oil was added to each well. Bacteria were incubated at 37°C, with shaking for 10 s and a 5-s pause before each reading, for 48 h. Controls included wells containing microbial cells with BHI broth alone (untreated). Growth was assessed by the OD_600_ every 30 min using a microplate reader (Bioscreen C; Growth Curves USA) (66). For CFU determinations, an aliquot of 100 μl of a suspension of bacterial cells was transferred to a microcentrifuge tube containing 900 μl of PBS. Finally, 2-fold serial dilutions of the suspensions were then performed, and aliquots of 100 μl from each dilution were plated onto BHI agar plates.

### Adhesion assay.

To investigate the role of METH, sucrose, or the combination in S. mutans adhesion to a solid surface, 200 μl of a suspension of 10^6^ bacteria in BHI broth alone or with 25 μM METH (Sigma), 2% sucrose (Sigma), or 25 μM METH plus 2% sucrose was added to individual wells of polystyrene 96-well microtiter plates (Corning) and incubated at 37°C in a 5% CO_2_ aerobic atmosphere. Bacteria were allowed to adhere to the bottom of the wells for 4 h. Following the adhesion stage, the wells with attached S. mutans cells were washed three times with PBS to remove nonadhered streptococci. Next, for CFU determination processing, 200 μl of trypsin was added to each well for 1 min to detach microbial cells from the plastic substrate, and an aliquot of 100 μl of a suspension of dissociated cells was transferred to a microcentrifuge tube containing 900 μl of PBS. Finally, 2-fold serial dilutions of the suspensions were then performed, and aliquots of 100 μl from each dilution were plated onto BHI agar (BD) plates. For fluorescence microscopy, S. mutans was labeled with fluorescein isothiocyanate (FITC) and adhered for 4 h at 37°C, the medium was gently aspirated, and bacteria were fixed with 2.5% glutaraldehyde (Sigma) for 1 h and washed three times with PBS. A coverslip was mounted by using a solution of 50% glycerol (Sigma) and 0.1 M *n*-propyl gallate (Sigma) in PBS. Samples were directly visualized with an upright Olympus AX41 microscope with fluorescence filters attached. Images of S. mutans adhesion to the polystyrene substrate were recorded with an Olympus DP70 camera and processed with Olympus DPC software. These experiments were performed in triplicate.

### Biofilm formation.

Two hundred microliters of a suspension with 10^6^ S. mutans cells in BHI broth alone or with 25 μM METH, 2% sucrose, or 25 μM METH plus 2% sucrose was added to individual wells of polystyrene 96-well plates. The plates were incubated at 37°C in a 5% CO_2_ aerobic atmosphere, and biofilms were formed over 48 h. The medium was gently aspirated, and biofilms were gently washed three times with 200 μl of PBS to remove nonadhered bacterial cells. Streptococci that remained attached to the plastic surface were considered true biofilms. All assays were carried out in triplicate.

### Quantification of biofilms.

Measurement of S. mutans biofilm formation was performed by CFU determinations and the 2,3-bis-(2-methoxy-4-nitro-5-sulfophenyl)-5-[(phenylamino)carbonyl]-2H-tetrazolium hydroxide (XTT) (Sigma) reduction assay. CFU determinations and the XTT reduction assay measure cell viability and the metabolic activity of the cells within biofilms, respectively.

**(i) CFU determinations.** Mature streptococcal biofilms were scraped from the bottom of each well using mechanical force with a 200-μl pipette tip, and a 1-ml suspension was transferred to a 2-ml microcentrifuge tube and sonicated to detach the cells (67). Briefly, the sonicator microtip was inserted into each microcentrifuge tube, and the biofilm-derived cells were sonicated for 8 s at 40% power. During the sonication process, each microcentrifuge tube was kept on ice to reduce the possibility of bacterial death due to an increase in temperature. An aliquot of 100 μl of the dissociated cell suspension was transferred to a microcentrifuge tube containing 900 μl of PBS. The suspension was then gently homogenized. Twofold serial dilutions of the suspensions were then performed, and aliquots of 100 μl from each dilution were plated onto BHI agar plates. To verify the impact of the sonication procedure on cell viability, we performed viable counts on separate cultures of S. mutans biofilm-derived cells before and after sonication. We found only a 5% reduction in the sonicated streptococci in biofilm-derived preparations.

**(ii) XTT reduction assay.** Aliquots of 50 μl of an XTT salt solution (1 mg/ml in PBS) and 4 μl of a menadione solution (1 mM in acetone; Sigma) were added to each well of a microtiter plate containing biofilms. Microtiter plates were incubated at 37°C for 5 h. The electron transport system in the cellular membrane of live bacteria reduces XTT tetrazolium salt to XTT formazan, resulting in a colorimetric change that correlates with cell viability (68). The colorimetric change was measured using a microtiter plate reader (Bio-Tek) at 492 nm. In all the experiments, microtiter wells containing heat-killed S. mutans and minimal medium alone were included as negative controls.

### Crystal violet staining.

We used the crystal violet method to stain the streptococcal biofilms on the plastic surface (69). Each well containing S. mutans biofilms was stained with 125 μl of a 0.1% solution of crystal violet (Sigma) for 15 min. Microtiter wells were rinsed three times with distilled water (dH_2_O), flipped over, tapped vigorously on a stack of paper towels to remove all excess dye, and air dried overnight. Next, a 125-μl suspension of 30% acetic acid (Thermo Fisher) in dH_2_O was added to each well to solubilize the crystal violet, followed by a 15-min incubation at room temperature. Finally, a 100-μl suspension of solubilized crystal violet was transferred to a clean microtiter plate and measured in a microtiter reader at 550 nm using 30 % acetic acid in dH_2_O as a negative control.

### Confocal microscopy.

The architecture of biofilms was examined using the Live/Dead biofilm viability kit (Invitrogen) and confocal microscopy. Briefly, S. mutans biofilms were grown for 48 h in 35-mm glass-bottom culture dishes (MatTek Corp.), alone or with 25 μM METH, 2% sucrose, or the combination of 25 μM METH and 2% sucrose; rinsed three times with PBS; and incubated for 30 min at room temperature in 2 ml of dH_2_O containing the fluorescent stain SYTO9 (6 μl; excitation wavelength, 500 nm; emission wavelength, 535 nm), with protection from light. The dishes were then rinsed three times with dH_2_O to remove excess stain. Microscopic examinations of biofilms formed in glass-bottom plates were performed using an upright Leica TCS SP5 confocal laser scanning microscope. To determine the structure and thickness of the biofilms, a series of horizontal (*x-y*) optical sections with a thickness of 1.175 μm were taken throughout the full length of the biofilm using a 63× objective. Confocal images of green fluorescence were recorded simultaneously using a multichannel mode. *z*-stack images and measurements were corrected by utilizing Leica LASX software in the deconvolution mode.

### METH injection, sucrose administration, and oral infection model.

METH users initially use small amounts of the drug intermittently before progressively increasing the dose (70). To simulate this pattern, we used increasing daily doses (2.5, 5, and 10 mg/kg/day on weeks 1, 2, and 3, respectively) of METH that were intraperitoneally (i.p.) administered to male/female C57BL/6 mice (6 to 8 weeks old; Charles River) over 21 days, as described previously (24). Animals that received equivalent volumes of PBS were used as controls. Mice that received the daily injection of METH lost approximately 2 g of body weight, compared with control mice (24). Two groups of mice, PBS and METH treated, were supplemented with 2% sucrose in the drinking dH_2_O. Animal dH_2_O consumption was monitored and recorded throughout the experiment. At day 21, METH- and PBS-treated C57BL/6 mice were anesthetized (100 mg/kg ketamine [Keta-set; Henry Schein] and 10 mg/kg xylazine [Anased; Henry Schein]), and a 50-μl suspension containing 10^7^ S. mutans strain Clarke 25175 bacteria in PBS was inoculated into each mouse mouth. Uninfected METH- or PBS-treated mice were used as additional controls. Animals were euthanized after 4 h (adhesion) or 24 h (biofilm), and teeth were carefully extracted to perform scanning electron microscopy (SEM), crystal violet staining, and CFU determinations. All animal studies were conducted according to the experimental practices and standards approved by the Institutional Animal Care and Use Committee (IACUC) at NYIT COM (protocol number 11-3). The IACUC at NYIT COM approved this study.

### Scanning electron microscopy.

To assess biofilm formation *in vivo* in the setting of METH administration, SEM was used to examine the teeth of untreated animals and animals treated with 25 μM METH, 2% sucrose, and 25 μM METH plus 2% sucrose. After extraction, teeth were fixed overnight (4% formaldehyde [Sigma] and 1% glutaraldehyde [Sigma] in PBS), washed for 5 min in PBS, and placed in 1% osmium tetroxide (Sigma) for 30 min. After a series of alcohol washes, the samples were critical-point dried (Samdri-790; Tousimis), mounted, gold coated (Desk-1; Denton Vacuum, Inc.), and viewed in a JEOL JSM-6400 scanning electron microscope in high-vacuum mode at 10 kV.

### CFU determinations in murine teeth.

Each murine tooth was held with a tweezer (Thermo Fisher) and carefully scraped using mechanical force with a 200-μl pipette tip in 1 ml PBS on a 2-ml microcentrifuge tube (Thermo Fisher). Next, the suspension containing the biofilm-derived cells was sonicated to detach the cells as described previously (67). Finally, serial dilutions of the sonicated suspension and plating on BHI agar dishes were performed as described above and incubated at 37°C for 24 h. Quantification of viable bacterial cells on teeth was performed by CFU counts.

### RNA extraction and cDNA synthesis.

For RNA extraction, S. mutans cells were suspended at a density of 5 × 10^8^ cells in 5 ml of PBS and homogenized with 0.5-mm-diameter zirconium-silica glass beads (Thermo Fisher) using a beater for 4 min to ensure complete lysis. Cell debris was removed by centrifugation at 10,000 rpm for 10 min at room temperature. RNA extraction was performed using the TRIzol Max bacterial RNA isolation kit (Thermo Fisher), according to the manufacturer’s instructions. To remove any genomic DNA carryover, the samples were treated with DNase I (Qiagen) for 30 min at 37°C, followed by heat inactivation for 5 min at 65°C. Next, 1 μg of total RNA was used to synthesize cDNA with the Bio-Rad iScript reverse transcriptase kit, according to the manufacturer’s instructions. The control reaction was set up using all components of the reaction mixture but without the reverse transcriptase enzyme (i.e., no reverse transcriptase).

### Real-time PCR.

The S. mutans glucosyltransferase genes selected for quantification were *gtfB* (71), *gtfC* (72), and *gtfD* (73), all involved in glycan metabolism and contributors to the cariogenicity of dental biofilms. The primers used for RT-PCR analysis are described in Table 1. The efficiency of each primer was tested by using a 10-fold serial dilution of the cDNA mixture, and only primers with efficiencies of between 95% and 105% were used for the analysis. The expression of genes was determined by RT-PCR using iQ SYBR green supermix (Bio-Rad). Two different control reactions were included in the analysis, i.e., a no-template control and a no-reverse-transcriptase control. We used 16S rRNA as a reference gene (Table 1). Relative expression was determined using the cycle threshold (ΔΔ*C_T_*) method on a Mastercycler RealPlex2 system (Eppendorf). Reactions were set up using 300 nM primers and 5 μl of the cDNA template (diluted 1:10). The cycling conditions used were as follows: 55°C for 30 min and then 40 amplification cycles of 95°C for 15 s, 55°C for 30 s, and 72°C for 30 s. The samples were cooled to 55°C, and a melting curve for temperatures between 55°C and 95°C, with 0.5°C increments, was recorded. All reactions were carried out in triplicate. Target gene expression was measured using expression relative to that of the 16S rRNA reference gene. Data analysis was carried out using Mastercycler ep RealPlex software (Eppendorf).

**TABLE 1:** Primer used in this study

Gene	Forward primer sequence (5′–3′)	Reverse primer sequence (5′–3′)
*gtfB*	AGCAATGCAGCCAATCTACAAAT	ACGAACTTTGCCGTTATTGTCA
*gtfC*	GGTTTAACGTCAAAATTAGCTGTATTAGC	CTCAACCAACCGCCACTGTT
*gtfD*	ACAGCAGACAGCAGCCAAGA	ACTGGGTTTGCTGCGTTTG
16S rRNA	CCTACGGGAGGCAGCAGTAG	CAACAGAGCTTTACGATCCGAAA

### pH measurement of S. mutans cultures.

The pH values of supernatants harvested from 24-h-old S. mutans cultures inoculated with 10^6^ bacteria in 25 ml of BHI broth alone or with 25 μM METH, 2% sucrose, or 25 μM METH plus 2% sucrose were measured using a calibrated pH meter (Thermo Fisher). Briefly, we placed the tip of the flat pH electrode on the surface of a stirring S. mutans culture in BHI broth to measure the pH. After measurement of a sample, the tip of the electrode was extensively rinsed with 10% bleach first, followed by dH_2_O, and blot dried with a soft tissue (Kimwipes; Kimberly-Clark). Sterile BHI broth alone or supplemented with METH, sucrose, or the combination was used as a baseline control.

### Lactic acid production determinations.

S. mutans culture supernatants were collected by centrifugation at 5,000 rpm for 10 min at 4°C. Using a 24-well plate, the following components were added individually to each well: 1 ml of dH_2_O, 1 ml of l-glutamic acid, 200 μl of NAD, and 20 μl of glutamate pyruvate transaminase. One hundred microliters of each supernatant was added to the sample wells, with the exception of the wells containing the negative controls. The plate was mixed, and absorbance 1 (A1) was read. Next, 20 μl of d-lactate dehydrogenase was added, and absorbance 2 (A2) was read. Finally, 20 μl of an l-lactate dehydrogenase solution was added, and the absorbance (A3) was read (r-biopharm kit; Boehringer Mannheim, Germany). All absorbances were spectrophotometrically measured at 340 nm (Bio-Tek Synergy LX).

### Oral rinse treatment *in vivo*.

Chlorhexidine (CHX) gluconate is a broad-spectrum germicidal oral rinse most widely used to treat plaque and gingivitis (74, 75). Adequate treatment with CHX has been shown to prevent S. mutans biofilm formation and cause bacterial detachment from teeth (76). Thus, after S. mutans infection, mice were anesthetized daily as described above, and each mouse mouth was pipetted three times with 100 μl of PBS or oral rinse containing 0.12% CHX gluconate (Peridex). On day 7 posttreatment, rodents were euthanized, and crystal violet staining was performed on the teeth to determine the S. mutans biomass. Photographs were taken with a Canon EOS Rebel T3 camera, and microbial biofilm on teeth was assessed using ImageJ 1.52e software (NIH).

### Surface preconditioning with human saliva.

The effect of surface human saliva preconditioning on S. mutans biofilm formation was examined. Saliva donors were instructed to avoid eating for 3 h or antimicrobial oral hygiene products prior to saliva collection. Saliva production by the donors was not stimulated. Microtiter plates were preconditioned with 100 μl of PBS or freshly collected unfiltered human saliva from a single donor and incubated at room temperature for 1 h. Wells were then aspirated, and the adsorbed conditioning film was washed once in sterile dH_2_O. Two hundred microliters of a suspension with 10^6^ bacteria in BHI broth alone or with 25 μM METH, 2% sucrose, or 25 μM METH plus 2% sucrose was individually dispensed into three wells of a 96-well microtiter plate and incubated for 24 h at 37°C in a 5% CO_2_ aerobic atmosphere. After incubation, the wells were aspirated and washed three times in sterile PBS, and biofilm formation was measured by the XTT reduction assay, CFU determinations, and crystal violet staining. Confocal microscopy was used to visualize the biofilm architectures and document their thickness. The protocol for the collection of human saliva was approved by the University of Texas at El Paso (UTEP) institutional review board (approval number 1200064-1).

### Statistical analysis.

All data were subjected to statistical analysis using Prism 8.0 (GraphPad Software). *P* values for multiple comparisons were calculated by analysis of variance (ANOVA) and were adjusted by the use of the Bonferroni correction. *P* values for individual comparisons were calculated using Student’s *t* test. *P* values of <0.05 were considered significant.
